# A smear test aiding with an automated slide reader acquires equivalent sensitivity to MGIT960 culture in pulmonary tuberculosis diagnosis

**DOI:** 10.1128/spectrum.03970-25

**Published:** 2026-06-26

**Authors:** Chao Zhang, Haoran Wang, Yuqing Chen, Adong Shen, Xia Yu, Hairong Huang

**Affiliations:** 1 National Clinical Laboratory on Tuberculosis, Beijing Key Laboratory for Drug Resistant Tuberculosis Research, Capital Medical University, Beijing Chest Hospital, Beijing Tuberculosis and Thoracic Tumor Institute, Beijing, China; 2 Kunming Children's Hospital, Kunming Medical University Affiliated, Kunming, Yunnan, China; Beijing Institute of Genomics, Chinese Academy of Sciences, Beijing, China; Capital Institute of Pediatrics, Beijing, China

**Keywords:** tuberculosis, smear microscopy, automated slide reader, artificial intelligence, diagnosis

## Abstract

Smear microscopy remains widely used for tuberculosis (TB) diagnosis due to its rapid turnaround and low cost, despite its relatively low sensitivity. With the advent of molecular tests, there is growing debate on whether the smear test remains relevant in clinical settings. An artificial intelligence (AI)-aided automated smear slide reader system (Zopomed, Zhengzhou, China) was evaluated at Beijing Chest Hospital. Sputum samples from suspected TB patients were used to prepare both Ziehl–Neelsen (ZN) and auramine O-stained slides. These were scanned by the automated reader under predefined field-of-view settings. Manual light-emitting diode fluorescence microscopy (for auramine O slides), MGIT960 culture, and Xpert MTB/RIF assay served as reference standards. Among 352 clinically confirmed TB patients, the detection rates were 18.75% by manual microscopy, 20.74% by automated ZN (1,000 fields), 32.67% by automated auramine O (300 fields), 30.68% by MGIT960 culture, and 42.05% by Xpert. Expanding the field of view significantly enhanced detection rates for both staining methods, with auramine O staining consistently outperforming ZN. The automated auramine O method (300 fields) identified 78.70% of culture-positive and 66.89% of Xpert-positive TB cases. All methods maintained specificities exceeding 99% when tested on 158 patients with non-mycobacterial pulmonary diseases. AI-aided auramine O smear testing represents a sensitive, rapid, and cost-effective approach to TB diagnosis. With the one-time investment in the automated system, this technique achieves sensitivity comparable to MGIT960 culture, offering a practical diagnostic solution for high-throughput clinical laboratories and resource-limited settings.

**IMPORTANCE:**

This study demonstrated that an AI-aided automated smear microscopy system, when used with auramine O-stained slides scanning 300 fields, achieves a sensitivity comparable to MGIT960 culture, while offering the advantages of rapid, same-day results and significant cost-effectiveness; this one-time investment in equipment, which is compatible with routine staining protocols, provides a highly practical and scalable solution to enhance tuberculosis diagnosis in both high-throughput laboratories and resource-limited settings.

## INTRODUCTION

Tuberculosis (TB) remains the leading cause of death caused by a single infectious pathogen worldwide (1). It represents the most common form of the disease (2), with a disproportionately high burden in low- and middle-income regions (3). Accurate and timely diagnosis is essential to interrupt disease transmission, underscoring the need for simple, cost-effective diagnostic tools. Conventional methods such as sputum smear microscopy and culture have served as cornerstone techniques for TB diagnosis for decades (4). Among these, smear microscopy—due to its rapidity, simplicity, and low cost—has long served as a frontline method for detecting acid-fast bacilli (AFB). Both Ziehl-Neelsen (ZN) and auramine O fluorescence staining are widely used for smear preparation, with the latter generally offering improved sensitivity (5–7). However, smear microscopy is limited by its relatively low sensitivity and operator-dependent results. Manual slide examination is not only time-consuming but also prone to reader fatigue, which may further reduce the detection rate of AFB.

In contrast to smear microscopy, culture-based methods offer significantly higher sensitivity. Both Löwenstein-Jensen (LJ) solid medium and MGIT960 liquid culture are widely used in clinical laboratories, with the latter demonstrating superior sensitivity. A key advantage of culture is its ability to detect viable bacilli, providing critical information about active infection. Nevertheless, long turn-around time (TAT) of culture, often takes weeks, diminishes its clinical utility for timely diagnosis. Additionally, the need for advanced biosafety facilities and technical expertise limits its applicability in peripheral healthcare settings. In recent years, nucleic acid amplification tests (NAATs), such as GeneXpert assay, have greatly improved the detection of bacteriological evidence (8, 9). Owing to their strong sensitivity, operational simplicity, and short TAT, the GeneXpert assay has been recommended as an initial test in TB diagnosis. However, the high cost of cartridges and equipment hinders their widespread adoption, particularly in resource-limited areas.

The advent of artificial intelligence (AI) might revive the outdated smear microscopy technique in TB diagnosis. Recent advances in AI-aided smear slide readers have demonstrated improved diagnostic sensitivity by automating image scanning and analysis, effectively emulating manual classification (10–12). There is growing consensus that AI-aided smear testing outperforms manual reading in sensitivity. These automated systems typically handle smear slides through automated loading, field navigation, focusing, and image capture, followed by algorithmic AFB identification and counting—ultimately replicating manual diagnostic workflows. By doing so, they can significantly enhance diagnostic sensitivity while saving time and reducing labor costs, though their real-world efficiency and stability require further validation.

In the present study, we firstly reported an AI-aided automated smear slide reader (Zopomed, Zhengzhou, China) equipped for both light microscopy and fluorescence microscopy, capable of reading ZN-stained and auramine O-stained slides prepared according to the routine standard operation protocol (SOP). This high-throughput system can process up to 200 slides per batch automatically. We assessed the compatibility of the device with both staining methods, examined how increasing the number of scanned fields improves sensitivity, and sought to identify an optimal diagnostic strategy. Furthermore, we compared the performance of this automated smear system with other commonly used laboratory methods—manual smear microscopy, MGIT960 culture, and Xpert assay—to clarify the role of this transformed conventional technique in an era increasingly dominated by molecular diagnostics for TB.

## MATERIALS AND METHODS

### Study design

Sputum samples were collected from suspected PTB patients in Beijing Chest Hospital from June to September 2023. Patients with symptoms suggestive of TB and abnormal radiographic findings were enrolled. Patients who were prescribed smear tests, MGIT960 culture, and Xpert assay on sputum for diagnostic purposes were enrolled continuously. Only one sample was included in this study if repeated samples from the same patient met the inclusion criteria. Besides the routine tests, only one extra smear slide was prepared with the residual sputum from routine work for each enrolled patient, and written consent from the patient was waived.

### Sputum smear staining and microscopy

Two smears were prepared for each sputum specimen and then stained either by the hot ZN technique (0.8% carbolfuchsin and 0.6% methylene blue) (#BA4011, Baso Company, China) or with 0.1% auramine O (#BA4070, Baso Company, China), counterstained with 0.5% potassium permanganate (#7722-64-7, Merck Company, Germany) for background quenching. According to the general algorithm, the auramine O-stained slides were viewed via light-emitting-diode (LED) fluorescence microscopy by experienced technicians, and the grading of the density of AFB on a slide was in accordance with the Union guidelines (13). Simultaneously, both ZN-stained and auramine O-stained smears were scanned by the automated smear slide reader system (Zopomed, Zhengzhou, China) (www.zopomed.com). For each automated ZN-stained slide, 300, 600, and 1,000 observed fields were set up for observation, which took 70, 130, and 210 s for the slide reader, respectively. For each automated auramine O-stained slide, 50, 150, and 300 observed fields were set up for observation, which took 30, 60, and 120 s for the slide reader, respectively.

### MGIT culture

*Mycobacterium* culture was performed with mycobacteria growth indicator tube (MGIT) 960 system (Becton, Dickinson and Company, USA) (14). Sputum samples were decontaminated with 1.5% *N*-acetyl-L- cysteine-NaOH (#616-91-1 & #1310-73-2, Merck Company, Germany) for 15 min at RT and neutralized with PBS buffer (#P1020, Solarbio Company, China). After centrifugation at 4,000 × *g* for 15 min, the sediments were resuspended in 2 mL PBS buffer. Then, 0.5 mL of this suspension was inoculated into the MGIT 960 tube, and then incubated in MGIT 960 system after supplementation of oleic acid-albumin-dextrose-catalase complex (OADC) (#212,351, BD Company, USA) and MGIT-PANTA (polymyxin B, amphotericin B, nalidixic acid, trimethoprim, and azlocillin).

### Xpert MTB/RIF assay

The Xpert MTB/RIF assay was performed according to the manufacturer’s instructions. Briefly, 1 mL of the sputum specimen was mixed with 2 mL of the sample reagent, vortexed for at least 10 s, and incubated for 15 min (RT). Then, 2 mL of the resulting mixture was transferred into the cartridge, loaded into GeneXpert (Cepheid Inc., Sunnyvale, CA, USA), and the detection procedure was initiated. Semiquantitative estimation of Mtb load was also determined by Xpert system as high, medium, low, very low, depending on cycle threshold (Ct) value and the positivity of the detected probes. Ct values of 15 to 18.9 indicated high, 19 to 24.9 indicated medium, 25 to 28.9 indicated low, and 29 to 40 indicated very low levels of bacteria.

### Statistical analysis

The statistical analysis was performed with GraphPad Prism 8.0.1 (GraphPad Software Inc., San Diego, CA, USA). The sensitivity, specificity, positive predictive value (PPV), and negative predictive value (NPV) were calculated, while the corresponding 95% CI was calculated using the Wilson score method. χ test (or χ test for trend) was used to determine the association of categorical variables, and McNemar’s test was used to compare the differences of various detection methods. Venn diagram was drawn using the website (https://bioinfogp.cnb.csic.es/tools/venny/index.html) to show the positive cases of different detection methods. Differences were considered statistically significant at *P* < 0.05.

## RESULTS

### Patient enrolled

A total of 530 TB suspects were enrolled, of whom 352 were diagnosed with PTB, 20 with non-tuberculous mycobacterial pulmonary disease (NTM-PD), and 158 with no evidence of mycobacterial infection. Among these cases, there were 317 males, accounting for 59.81% of the total. The median age was 57 years old (range, 13–90 years old).

### Diagnosis performance of the automated smear reader system

**Fig 1:**
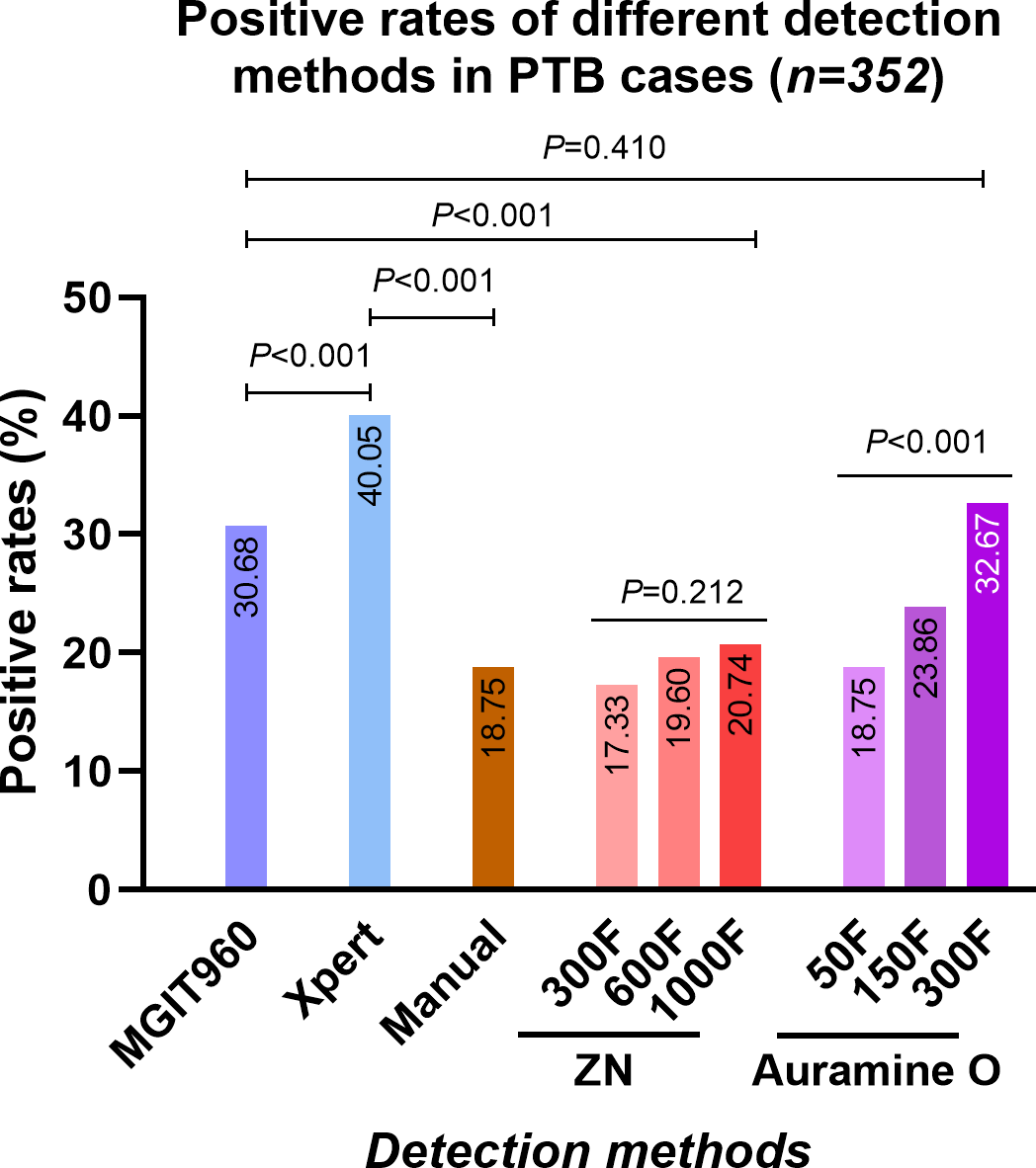
The positive rates of different detection methods in 352 PTB cases. The relationship between the fields of the automated smear microscopy system and its diagnostic performance was calculated using χ test for trend. The differences of positive rates among different detection methods were calculated using the McNemar’s test. F represents the detection fields of the automated smear microscopy system.

Based on clinical diagnosis as the reference, we found the positive rates among the manual microscopy, automated-auramine O staining method with 300 fields scanned, automated-ZN staining method with 1,000 fields scanned, MGIT960 culture, and Xpert assay were 18.75% (66/352), 32.67% (115/352), 20.74% (73/352), 30.68% (108/352), and 42.05% (148/352), respectively (Fig. 1; Table 1). Increasing the reading field number elevated the positive detection rates for both ZN-stained slides and auramine O-stained slides, but this tendency was much evident for auramine O-stained slides (*P* < 0.05, χ^2^ = 18.74). The positive rate of the Xpert was higher than that of all other detection methods (*P* < 0.05). The positive rate of the MGIT960 culture method was significantly higher than that of the automated-ZN staining method (*P* < 0.05), but it is similar to the detection rate of the automated-auramine O staining method (300 fields) (*P* > 0.05) (Fig. 1). All the tested methods acquired over 99% specificities among the 158 non-mycobacterial disease cases (Table 1).

**TABLE 1:** Diagnosis performance of different detection methods in detecting PTB (*n* = 510)^*a*^

Detection methods		Sensitivity (95% CI)	Specificity (95% CI)	PPV (95% CI)	NPV (95% CI)
Auramine O- Manual		18.75% (66/352, 15.02%–23.16%)	100.00% (158/158, 97.63%–100.00%)	100.00% (66/66, 94.50%–100.00%)	35.59% (158/444, 31.27%–40.14%)
Xpert		42.05% (148/352, 37.01%–47.24%)	100.00% (158/158, 97.63%–100.00%)	100.00% (148/148, 97.54%–100.00%)	43.65% (158/362, 38.67%–48.76%)
MGIT960		30.68% (108/352, 26.12%–35.62%)	100.00% (158/158, 97.63%–100.00%)	100.00% (108/108, 96.55%–100.00%)	39.30% (158/402, 34.66%–44.15%)
ZN-Auto	300F	17.33% (61/352, 13.66%–21.77%)	100.00% (158/158, 97.63%–100.00%)	100.00% (61/61, 93.94%–100.00%)	35.19% (158/449, 30.91%–39.75%)
	600F	19.60% (69/352, 15.72%–24.16%)	100.00% (158/158, 97.63%–100.00%)	100.00% (69/69, 94.57%–100.00%)	35.83% (158/441, 31.47%–40.45%)
	1000F	20.74% (73/352, 16.83%–25.28%)	100.00% (158/158, 97.63%–100.00%)	100.00% (73/73, 95.00%–100.00%)	36.16% (158/437, 31.79%–40.76%)
Au O-Auto	50F	18.75% (66/352, 15.02%–23.16%)	100.00% (158/158, 97.63%–100.00%)	100.00% (66/66, 94.50%–100.00%)	35.59% (158/402, 31.27%–40.14%)
	150F	23.86% (84/352, 19.71%–28.58%)	100.00% (158/158, 97.63%–100.00%)	100.00% (84/84, 95.63%–100.00%)	37.09% (158/426, 32.63%–41.77%)
	300F	32.67% (115/352, 27.98%–37.73%)	99.37% (157/158, 96.51%–99.89%)	99.14% (115/116, 95.28%–99.85%)	39.85% (157/394, 35.14%–44.75%)

^*a*^ Clinical diagnosis served as golden standard. F represents scanning fields of the automated smear scanner system. ZN-Auto and Au O-auto represent the automated microscope scanning of smears prepared by Ziehl-Neelsen and Auramine O staining methods, respectively.

**Fig 2:**
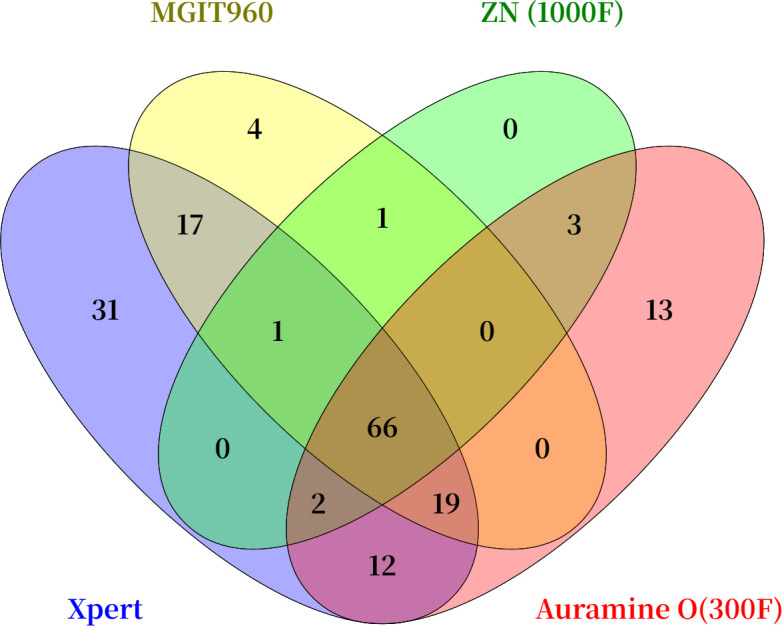
The Venn diagram of different detection methods in 352 PTB cases. Blue circle: cases detected by Xpert, yellow circle: cases detected by MGIT960, green circle: cases detected by automated smear microscopy stained by ZN, red circle: cases detected by automated smear microscopy stained by auramine-O.

According to a Venn diagram (Fig. 2), Xpert assay, MGIT960 culture, and automated-auramine O-300 fields detected 31, 4, and 13 extra positive cases by themselves, but none by automated-ZN-1,000 fields.

### The sensitivity of different smear tests among culture-positive TB cases

**Fig 3:**
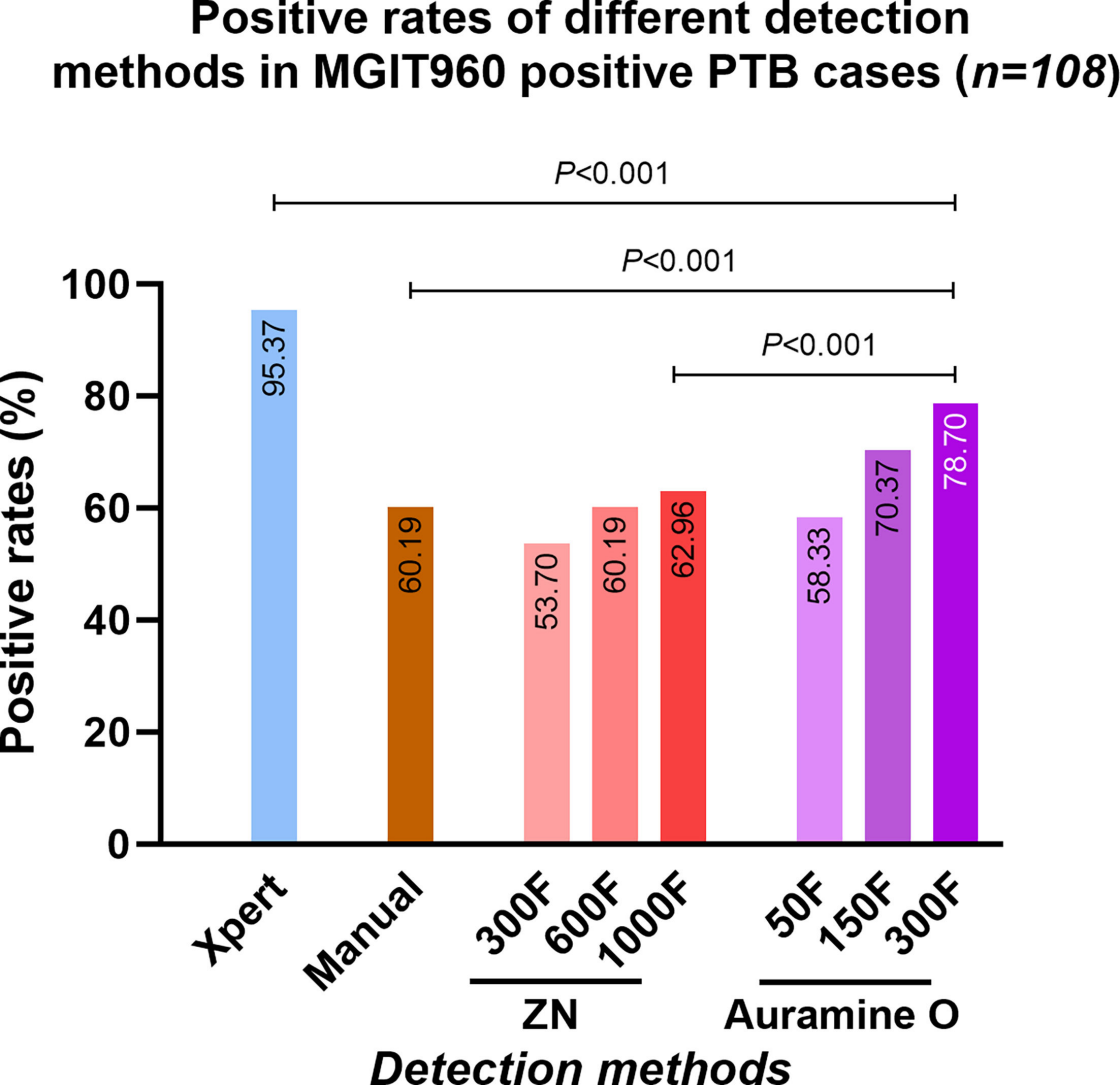
The positive rates of different detection methods in 108 MGIT960-positive PTB cases. The differences of positive rates among different detection methods were calculated using the McNemar’s test. F represents the detection fields of the automated smear microscopy system.

Among the 108 culture-positive specimens, the Xpert assay detected 95.37% of cases, followed by automated-auramine O (300 fields) (78.70%) (Fig. 3). The Xpert assay demonstrated a significantly higher detection rate than automated-auramine O (300 fields), but the latter was obviously more sensitive than manual smear test and automated-ZN (1,000 fields) (all *P* < 0.05). To investigate the discordance between culture and the automated reader, we analyzed the clinical characteristics of the 23 cases that were MGIT960-positive but yielded negative results by automated auramine O scanning (300 fields). These discordant cases were predominantly paucibacillary. According to Xpert MTB/RIF semi-quantitative grading, 6 cases (26.09%) were classified as “very low,” 11 cases (47.83%) as “low,” and 1 case (4.35%) as “medium” positive; notably, 5 cases (21.74%) were negative by the Xpert assay. This suggests that while the AI reader significantly boosts sensitivity, it may still miss cases with extremely low mycobacterial burdens that are below the detection limit of microscopy but viable in liquid culture.

### The sensitivity of different smear tests among Xpert-positive TB cases

**Fig 4:**
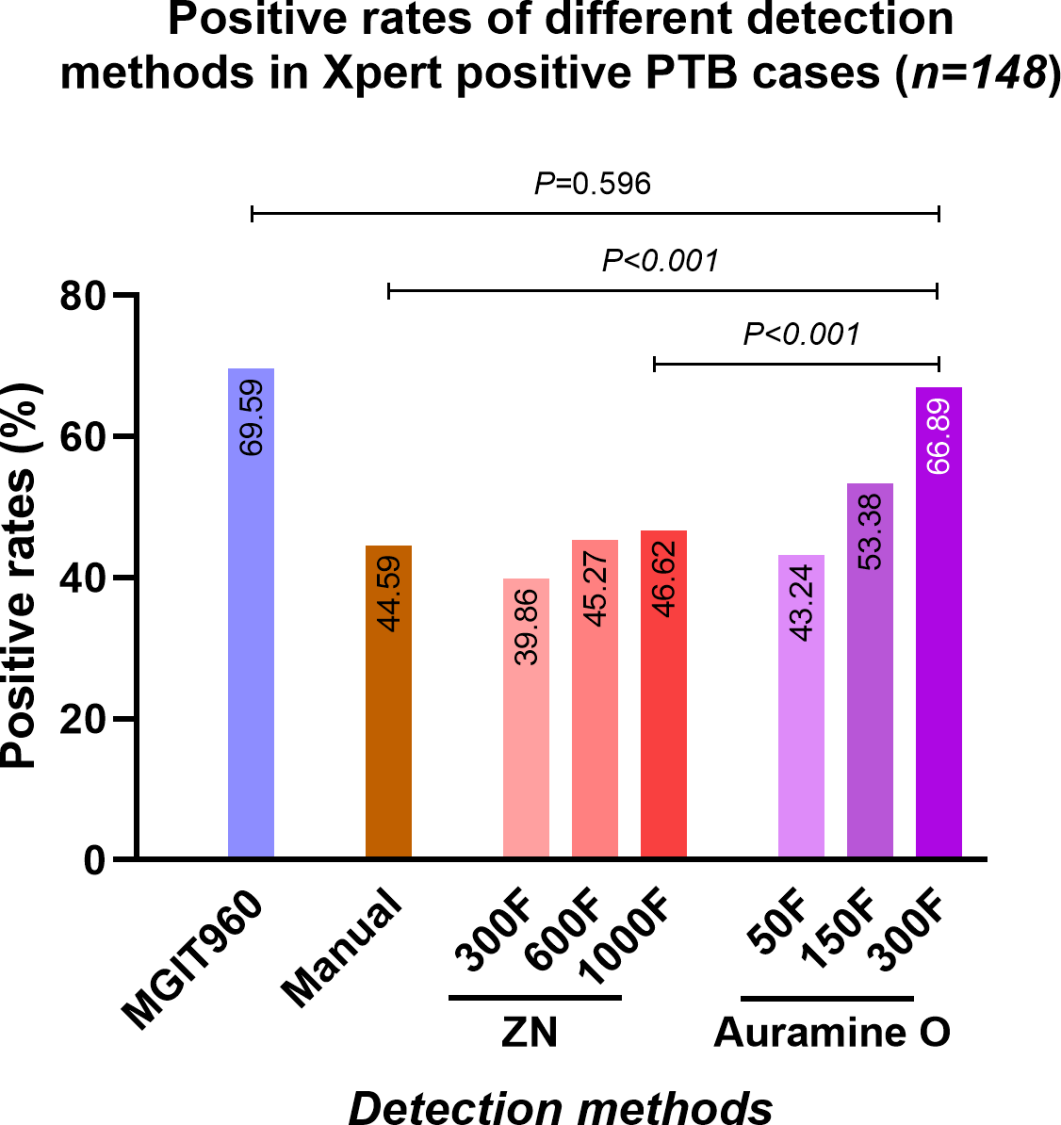
The positive rates of different detection methods in 148 Xpert-positive PTB cases. The differences of positive rates among different detection methods were calculated using the McNemar’s test. F represents the detection fields of the automated smear microscopy system.

Among the 148 Xpert-positive specimens, MGIT960 culture detected 69.59% of cases, followed by automated-auramine O (300 fields) (66.89%). Automated-auramine O (300 fields) acquired equivalent sensitivity to MGIT960 liquid culture (*P* = 0.596) but was significantly more sensitive than both manual smear test and automated-ZN (1,000 fields) (all *P* < 0.05) (Fig. 4).

### The positive detection outcomes of different methods across Xpert categories in 352 TB cases

The detection rates of different methods were analyzed by stratifying the Xpert outcomes according to its semi-quantitative levels (Table 2). For the specimens with high bacterial loads, both MGIT960 culture and automated-auramine O (200 and 300 fields) successfully detected nearly all cases. In contrast, the ZN-automated method generally yielded lower detection rates, even when 1,000 fields were observed. In the low-level positive categories, all the included methods manifested inferior detection rates, although MGIT960 culture and automated-auramine O (300 fields) exhibited relatively better performance compared with the other methods.

**TABLE 2:** The positive detection outcomes of different methods in different Xpert categories in 352 TB cases^*a*^

Index method		Xpert outcomes (%, *n*)				
		High (19)	Medium (46)	Low (46)	Very low (37)	Negative (204)
Auramine O-Manual		84% (16)	89% (41)	17% (8)	3% (1)	0% (0)
ZN- Auto	300F	79% (15)	76% (35)	17% (8)	3% (1)	1% (2)
	600F	84% (16)	83% (38)	24% (11)	5% (2)	1% (2)
	1000F	84% (16)	85% (39)	26% (12)	5% (2)	2% (4)
Au O-Auto	50F	95% (18)	83% (38)	15% (7)	3% (1)	1% (2)
	200F	100% (19)	91% (42)	33% (15)	8% (3)	3% (5)
	300F	100% (19)	98% (45)	61% (28)	19% (7)	8% (16)
MGIT960		100% (19)	100% (46)	63% (29)	24% (9)	3% (5)

^*a*^ F represents scanning fields of the automated smear scanner system. ZN-Auto and Au O-auto represent the automated microscope scanning of smears prepared by Ziehl-Neelsen and Auramine O staining methods, respectively.

## DISCUSSION

The past decade has witnessed significant breakthroughs in molecular diagnostic techniques for tuberculosis. Despite gradual reductions in the cost and complexity of these tests, they remain inaccessible to many peripheral laboratories and resource-limited regions with high TB burden, where affordable and practical diagnostic methods are urgently needed. In this study, we introduced an AI-aided smear reading system which requires only a one-time investment in equipment. Our results demonstrated that, with this automated reader, the smear test achieved sensitivity comparable to MGIT960 culture. This is of great significance, as MGIT960 culture necessitates costly instruments and consumables, in addition to its characteristically long TAT.

A study by Tan et al. evaluated an integrated smear test method involving multiple devices for smear preparation, ZN-staining, and slide scanning, which also reported equivalent sensitivity to MGIT960 culture (15). In contrast, our study only utilized a single slide reader but acquired comparable efficacy in AFB detection. Moreover, Tan’s system required a specific sputum container and involved manual transfer of slides between instruments (15), whereas our approach offers greater operational simplicity.

While some AI-aided microscopy systems, such as the TBDx (Signature Mapping Medical Sciences), have shown promise (16), the system evaluated here presents unique advantages in throughput and versatility. Unlike systems that process single slides or require specific cartridges, the Zopomed reader is a high-throughput device capable of loading 200 slides per batch, significantly reducing hands-on time for technicians in high-volume hospitals. Furthermore, its dual capability to scan both ZN and fluorescent-stained slides allows laboratories to transition from light to fluorescence microscopy without purchasing new automation platforms. While sensitivity is comparable across various high-end AI systems, the integration of batch processing and “walk-away” automation addresses the critical bottleneck of labor intensity that other semi-automated solutions have not fully resolved.

To our knowledge, this smear test aiding with an automated slide reader represents an improvement in sensitivity over conventional methods, as well as previously reported automated slide readers (17, 18). Besides, this approach also demonstrated high specificity in our evaluation. Results were highly reliable when a substantial number of AFB were detected. In cases with scanty AFB, however, misinterpretation remained possible. Since the system retains images of suspected AFB, such cases can be easily verified by manual review. It is worth noting that as the number of scanned fields increases, the frequency of scanty reports also rises, underscoring the importance of result verification to prevent diagnostic errors.

A major strength of this system is its compatibility with routine SOP for both ZN-staining and auramine O-staining in clinical laboratories. Both staining methods are simple and cost-effective, with manual smear preparation costing less than 1 US dollar per test. In our study, auramine O-staining yielded significantly higher detection rates than ZN staining. This is largely attributable to the larger field of view observable with fluorescence microscopy, whereas ZN staining requires oil immersion and is more time-consuming. Although increasing the number of observed fields improved detection rates for both methods, this also substantially prolongs the scanning time per slide—a limitation in high-volume laboratory settings. In laboratories with lower workloads, however, expanding the field number may help identify more TB cases. Notably, the slide reader can automatically terminate scanning once sufficient AFB are identified, providing rapid confirmation for samples with high bacterial loads within seconds—a feature that significantly enhances laboratory workflow efficiency.

The automated smear slide reader offers a compelling balance between cost and performance. The automated smear slide reader costs approximately $25,000–30,000 USD (market dependent), which is roughly one-fourth of the price of an MGIT960 system and significantly lower than the GeneXpert system modules. Regarding operational costs, smear consumables (slides and reagents) cost less than $1 USD per test, whereas MGIT tubes and supplements cost $10–15 USD, and Xpert cartridges are higher. Turnaround time (TAT) is another critical factor; the automated reader provides results in under 5 min per slide (210 s for 1,000 fields ZN; 120 s for 300 fields Auramine O), allowing for same-day diagnosis. In contrast, Xpert takes approximately 2 h, and MGIT960 culture requires an average of 10–21 days for positivity. Nevertheless, a key advantage of MGIT960 culture and GeneXpert system is its ability to yield isolates for subsequent drug susceptibility testing.

According to the Venn diagram in this study (Fig. 2), there was substantial overlap between cases detected by both methods. Among the discrepant results, 23 were MGIT960 culture-positive but automated auramine O-negative (300 fields), and 30 were vice versa. Thus, using the automated slide reader as a preliminary screening tool prior to culture could substantially reduce costs while still ensuring adequate strain recovery for drug resistance testing.

This study included 20 sputum samples from patients with nontuberculous mycobacterial (NTM) infections. These patients were not classified as non-TB, given the rising global incidence of NTM pulmonary disease (19, 20). Identifying NTM AFB is also of clinical significance, but due to the limitations of smear tests and cultures to differentiate Mtb from NTM, classifying these cases as non-TB would compromise the specificity of smear tests and cultures. In contrast, the Xpert assay was unaffected by this issue.

Several limitations should be noted in this study. First, regarding the study design, assays were performed on sputum samples collected from the same day but not from the same specimen aliquot (e.g., a split-sample design). While this reflects real-world clinical workflows where multiple samples are collected, the heterogeneity in bacterial load between different sputum contributions may introduce variability that attenuates the observed agreement between methods. Second, it was conducted in a single clinical center, and different clinical settings with varying TB prevalence and disease profiles may affect the detection rates of the methods evaluated. Third, we did not include a group with more than 300 reading fields for auramine O-stained slides. Fourth, although we highlight the low equipment cost, a formal health economic analysis calculating the incremental cost-effectiveness ratio (ICER) was not performed. Additionally, the long-term mechanical stability and maintenance requirements of the AI reader in high-dust or high-humidity settings require further longitudinal observation. Finally, a fundamental limitation of smear microscopy (including this AI-aided system) is its inability to differentiate MTB from NTM. Given the increasing detection rates observed with more fields, we would expect even higher yields with greater numbers of fields, potentially bringing the sensitivity closer to that of Xpert assay. This hypothesis warrants further investigation.

### Conclusion

This study demonstrates that an AI-aided smear slide reading system significantly improves AFB detection sensitivity, achieving performance comparable to MGIT960 culture. With a one-time investment in the equipment, smear microscopy becomes a low-cost, rapid, and sensitive diagnostic tool, offering a practical solution for high-throughput clinical laboratories and resource-limited settings where TB diagnostics remain a challenge.

## Supplementary Material

Reviewer comments

## Data Availability

The data of this study are available from the corresponding author upon reasonable request.

## SUPPLEMENTAL MATERIAL

OPEN PEER REVIEW reviewer-comments.pdf An accounting of the reviewer comments and feedback.

ASM does not own the copyrights to Supplemental Material that may be linked to, or accessed through, an article. The authors have granted ASM a non-exclusive, world-wide license to publish the Supplemental Material files. Please contact the corresponding author directly for reuse.
