# Supplementary material for: A smear test aiding with an automated slide reader acquires equivalent sensitivity to MGIT960 culture in pulmonary tuberculosis diagnosis
Source: Microbiol Spectr. 2026 Jun 26;14(8):e03970-25. doi: 10.1128/spectrum.03970-25 (PMC13436338; doi:10.1128/spectrum.03970-25)
Supplement: Supplementary file 1 [file reviewer-comments.pdf]

AMERICAN SOCIETY FOR MICROBIOLOGY | Microbiology Spectrum

A Smear Test Aiding with an automated slide reader acquires equivalent sensitivity to MGIT960 culture in Pulmonary Tuberculosis Diagnosis

Chao Zhang, Haoran Wang, Yuqing Chen, Adong Shen, Xia Yu, and Hairong Huang

Corresponding Author(s): Hairong Huang, Beijing chest hospital

Review Timeline:

Submission Date:	December 10, 2025
Editorial Decision:	February 8, 2026
Revision Received:	February 14, 2026
Accepted:	March 7, 2026

Editor: Fei Chen

Reviewer(s): Disclosure of reviewer identity is with reference to reviewer comments included in decision letter(s). The following individuals involved in review of your submission have agreed to reveal their identity: Yi Wang (Reviewer #1)

Transaction Report:

(Note: With the exception of the correction of typographical or spelling errors that could be a source of ambiguity, letters and reports are not edited. The original formatting of letters and referee reports may not be reflected in this compilation.)

DOI: https://doi.org/10.1128/spectrum.03970-25

Re: Spectrum03970-25 (**A Smear Test Aiding with an automated slide reader acquires equivalent sensitivity to MGIT960 culture in Pulmonary Tuberculosis Diagnosis**)

Dear Dr. Hairong Huang:

Thank you for the privilege of reviewing your work. Below you will find my comments, instructions from the Spectrum editorial office, and the reviewer comments.

Please return the manuscript within 60 days; if you cannot complete the modification within this time period, please contact me. If you do not wish to modify the manuscript and prefer to submit it to another journal, notify me immediately so that the manuscript may be formally withdrawn from consideration by Spectrum.

Revision Guidelines
To submit your modified manuscript, log into the submission site at https://spectrum.msubmit.net/cgi-bin/main.plex. Go to Author Tasks and click the appropriate manuscript title to begin. The information you entered when you first submitted the paper will be displayed; update this as necessary. Note the following requirements:

• Upload point-by-point responses to the issues raised by the reviewers in a file named "Response to Reviewers," NOT in your cover letter.
• Upload a compare copy of the manuscript (without figures) as a "Marked-Up Manuscript" file.
• Upload a clean .DOC/.DOCX version of the revised manuscript and remove the previous version.
• Each figure must be uploaded as a separate, editable, high-resolution file (TIFF or EPS preferred), and any multipanel figures must be assembled into one file.
• Any supplemental material intended for posting by ASM should be uploaded with their legends separate from the main manuscript. You can combine all supplemental material into one file (preferred) or split it into a maximum of 10 files with all associated legends included.

For complete guidelines on revision requirements, see our Submission and Review Process webpage. Submission of a paper that does not conform to guidelines may delay acceptance of your manuscript.

Data availability: ASM policy requires that data be available to the public upon online posting of the article, so please verify all links to sequence records, if present, and make sure that each number retrieves the full record of the data. If a new accession number is not linked or a link is broken, provide Spectrum production staff with the correct URL for the record. If the accession numbers for new data are not publicly accessible before the expected online posting of the article, publication may be delayed; please contact production staff (Spectrum@asmusa.org) immediately with the expected release date.

Publication Fees: For information on publication fees and which article types are subject to charges, visit ourwebsite. If your manuscript is accepted for publication and any fees apply, you will be contacted separately about payment during the production process; please follow the instructions in that e-mail. Arrangements for payment must be made before your article is published.

ASM Membership: Corresponding authors may join or renew ASM membership to obtain discounts on publication fees. Need to upgrade your membership level? Please contact Customer Service at Service@asmusa.org.

The ASM Journals program strives for constant improvement in our submission and publication process. Please tell us how we can improve your experience by taking this quick Author Survey.

Thank you for submitting your paper to Spectrum.

Sincerely,
Fei Chen
Editor
Microbiology Spectrum

Reviewer #1 (Comments for the Author):

This manuscript presents a valuable evaluation of an AI-aided automated slide reader system for pulmonary tuberculosis diagnosis. The authors compare the performance of this system using both Ziehl-Neelsen and Auramine O staining against established reference standards like MGIT960 culture and Xpert MTB/RIF. The finding that the automated Auramine O method achieves sensitivity comparable to liquid culture is clinically significant, particularly for high-throughput and resource-limited settings where culture is not feasible. The study design is generally sound, the sample size is adequate, and the data supports the primary conclusions. However, there are inconsistencies in the reported statistical values between the Abstract, Results, and

Tables that must be rectified. Additionally, some minor formatting errors and punctuation need to be polished to enhance the readability of the article before publication.

1. All assays were performed on sputum collected from the same patient but not from the same specimen aliquot. This design may introduce variability that attenuates observed agreement. Please discuss the potential impact on sensitivity comparisons.
2. Among culture-positive cases, 23 were negative by automated auramine-O (300 fields). No clinical characteristics (bacterial load, cavitation, HIV status, treatment history) of these discrepant results are shown. A short table or narrative analysis should be added to identify potential predictors of false negativity.
3. The Discussion would benefit from a short paragraph comparing Zopomed with other commercial AI smear systems in terms of hardware cost, throughput and validated sensitivity.
4. Expand the Limitations section to include the absence of a cost-effectiveness analysis and the lack of long-term stability data for the AI reader.
5. Data Inconsistency:
There is a discrepancy in the reported detection rate (positive rate) for the Xpert assay across different sections of the manuscript.
In the Abstract (Line 32), it is stated as 40.95%.
In the Results (Line 180), it is listed as 40.05% (148/352).
However, 148 divided by 352 is approx. 42.05%, which matches the sensitivity value listed in Table 1.
Please calculate the correct percentage and ensure this value is consistent throughout the Abstract, Results text, and Tables.
6. Formatting and Punctuation:
Page 9, Line 174: There is a punctuation error in the phrase "(ranged ,13-90 years old)". The comma is misplaced.
Table 1: The text alignment in the "Detection methods" column is broken and difficult to read (e.g., "ZN-au" / "tomat" / "ed" are on separate lines, and "Auram" / "ine" are separated). Please adjust the column width or hyphenation to ensure the method names are displayed correctly.

Reviewer #2 (Comments for the Author):

The article evaluated the clinical performance of an AI-aided automated smear slide reader. The AI-aided method demonstrated increased sensitivity of smear testing, offering a practical diagnostic solution for high-throughput clinical laboratories and resource-limited settings.
1.Data inconsistency between Table 1 and the Venn diagram: In Table 2, there are 11 Xpert-negative patients with positive Auramine O results (300 fields). However, the Venn diagram (Figure 2) shows 16 such cases (13 exclusively Auramine O-positive plus 3 overlapping with ZN only). The authors should clarify and reconcile this discrepancy.
2.Limitation regarding NTM differentiation: The smear test cannot differentiate MTB from NTM. This important limitation should be explicitly emphasized in the Limitations section, given the rising global incidence of NTM pulmonary disease.
3.Cost-effectiveness and turnaround time: The Discussion mentions cost-effectiveness comparisons among different methods. It is recommended to provide specific cost estimates (equipment, consumables, maintenance) and actual turnaround times for each method to support these claims.
4.Table 1 enhancement: The number of cases (n) in each group should be clearly listed in Table 1 to facilitate interpretation of sensitivity, specificity, and predictive values.
5.Comparison with similar AI-aided methods: The Discussion should be expanded to include a more comprehensive comparison with other AI-aided smear reading systems from recent studies, highlighting the unique advantages and potential limitations of the current system relative to existing technologies.

This manuscript presents a valuable evaluation of an AI-aided automated slide reader system for pulmonary tuberculosis diagnosis. The authors compare the performance of this system using both Ziehl–Neelsen and Auramine O staining against established reference standards like MGIT960 culture and Xpert MTB/RIF. The finding that the automated Auramine O method achieves sensitivity comparable to liquid culture is clinically significant, particularly for high-throughput and resource-limited settings where culture is not feasible. The study design is generally sound, the sample size is adequate, and the data supports the primary conclusions. However, there are inconsistencies in the reported statistical values between the Abstract, Results, and Tables that must be rectified. Additionally, some minor formatting errors and punctuation need to be polished to enhance the readability of the article before publication.

1. All assays were performed on sputum collected from the same patient but not from the same specimen aliquot. This design may introduce variability that attenuates observed agreement. Please discuss the potential impact on sensitivity comparisons.
2. Among culture-positive cases, 23 were negative by automated auramine-O (300 fields). No clinical characteristics (bacterial load, cavitation, HIV status, treatment history) of these discrepant results are shown. A short table or narrative analysis should be added to identify potential predictors of false negativity.
3. The Discussion would benefit from a short paragraph comparing Zopomed with other commercial AI smear systems in terms of hardware cost, throughput and validated sensitivity.
4. Expand the Limitations section to include the absence of a cost-effectiveness analysis and the lack of long-term stability data for the AI reader.
5. Data Inconsistency:

There is a discrepancy in the reported detection rate (positive rate) for the Xpert assay across different sections of the manuscript.

In the Abstract (Line 32), it is stated as 40.95%.

In the Results (Line 180), it is listed as 40.05% (148/352).

However, 148 divided by 352 is approx. 42.05%, which matches the sensitivity value listed in Table 1.

Please calculate the correct percentage and ensure this value is consistent throughout the Abstract, Results text, and Tables.

6. Formatting and Punctuation:

Page 9, Line 174: There is a punctuation error in the phrase "(ranged ,13-90 years old)". The comma is misplaced.

Table 1: The text alignment in the "Detection methods" column is broken and difficult to read (e.g., "ZN-au" / "tomat" / "ed" are on separate lines, and "Auram" / "ine" are separated). Please adjust the column width or hyphenation to ensure the method names are displayed correctly.

Dear Editor and Reviewers,

We would like to thank you for the constructive comments and suggestions concerning our manuscript entitled “A Smear Test Aiding with an automated slide reader acquires equivalent sensitivity to MGIT960 culture in Pulmonary Tuberculosis Diagnosis”. These comments have been very helpful in revising and improving our paper. We have carefully studied the comments and have made corrections which we hope meet with approval.

Below is our point-by-point response to the reviewers’ comments:

Reviewer #1

Comment 1: All assays were performed on sputum collected from the same patient but not from the same specimen aliquot. This design may introduce variability that attenuates observed agreement. Please discuss the potential impact on sensitivity comparisons.

Response: Thanks for your suggestions! We agree with the reviewer that using different sputum samples rather than a homogenized split-sample design introduces biological variability. In the revised manuscript, we have explicitly added this to the Limitations section in the Discussion (Line 313-315). We acknowledged that the heterogeneity in bacterial load between samples might attenuate the observed agreement and potentially underestimate the sensitivity of the automated system.

Comment 2: Among culture-positive cases, 23 were negative by automated auramine-O (300 fields). No clinical characteristics (bacterial load, cavitation, HIV status, treatment history) of these discrepant results are shown. A short table or narrative analysis should be added to identify potential predictors of false negativity.

Response: We appreciate this insightful suggestion. We have added a narrative analysis of these 23 discrepant cases in the Results section (Line 201-210). Our analysis indicated that these cases were predominantly paucibacillary (low bacterial load in Xpert), suggesting that extremely low bacterial load remains the primary predictor for false negativity in the automated smear system.

Comment 3: The Discussion would benefit from a short paragraph comparing

Zopomed with other commercial AI smear systems in terms of hardware cost, throughput and validated sensitivity.

Response: Thanks for your suggestions! We have expanded the Discussion section to include a comparison with other AI-aided systems (Line 248-259). We highlighted that while sensitivity is comparable to other emerging technologies, the unique advantage of the evaluated system lies in its high-throughput design (200 slides/batch) and dual-modality (ZN and Fluorescence) capability, which distinguishes it from manual digital microscopes or single-slide scanners.

Comment 4: Expand the Limitations section to include the absence of a cost-effectiveness analysis, lack of long-term stability data for the AI reader.

Response: Thanks for your suggestions! We have significantly revised the Limitations section (Line 323-327). We added statements clarifying that: 1) a formal health economic analysis was not performed; 2) long-term mechanical stability in varying environments requires further observation.

Comment 5: Data Inconsistency: There is a discrepancy in the reported detection rate (positive rate) for the Xpert assay across different sections of the manuscript. In the Abstract (Line 32), it is stated as 40.95%. In the Results (Line 180), it is listed as 40.05% (148/352). However, 148 divided by 352 is approx. 42.05%, which matches the sensitivity value listed in Table 1.Please calculate the correct percentage and ensure this value is consistent throughout the Abstract, Results text, and Tables.

Response: We are sorry about this mistake. We have corrected this inconsistency in Abstract, Results text and tables/figures, and we checked all the data in this study.

Comment 6: Formatting and Punctuation: Page 9, Line 174: There is a punctuation error in the phrase "(ranged ,13-90 years old)". The comma is misplaced. Table 1: The text alignment in the "Detection methods" column is broken and difficult to read (e.g., "ZN-au" / "tomat" / "ed" are on separate lines, and "Auram" / "ine" are separated). Please adjust the column width or hyphenation to ensure the method names are displayed correctly.

Response: Thanks for your suggestions! We have corrected the misalignment of punctuation marks, and abbreviated the Detection Methods in the Tables

(Table 1&2), so that all the method names can be presented correctly.

Reviewer #2

Comment 1: Data inconsistency between Table 1 and the Venn diagram: In Table 2, there are 11 Xpert-negative patients with positive Auramine O results (300 fields). However, the Venn diagram (Figure 2) shows 16 such cases (13 exclusively Auramine O-positive plus 3 overlapping with ZN only). The authors should clarify and reconcile this discrepancy.

Response: We are sorry about this mistake. After re-verifying and calculation, it turns out that 16 samples were Auramine O positive with Xpert-negative, we have corrected in the Table 2.

Comment 2: Limitation regarding NTM differentiation: The smear test cannot differentiate MTB from NTM. This important limitation should be explicitly emphasized in the Limitations section, given the rising global incidence of NTM pulmonary disease.

Response: Thanks for your suggestions! We have expanded in the Limitations section (Line 327-329) and acknowledged that a fundamental limitation of the smear test is its inability to distinguish Mtb from NTM, necessitating confirmatory testing.

Comment 3: Cost-effectiveness and turnaround time: The Discussion mentions cost-effectiveness comparisons among different methods. It is recommended to provide specific cost estimates (equipment, consumables, maintenance) and actual turnaround times for each method to support these claims.

Response: Thanks for your suggestions! We have updated the Discussion to provide more specific estimates (Line 285-296). We detailed that the automated reader equipment cost is approximately 25-30% of an MGIT system, with per-test consumable costs under $1 USD (vs. >$10 USD for culture/molecular tests). We also explicitly compared the TAT: <5 minutes for the automated smear versus 2 hours for Xpert and weeks for MGIT culture.

Comment 4: Table 1 enhancement: The number of cases (n) in each group should be

clearly listed in Table 1 to facilitate interpretation of sensitivity, specificity, and predictive values.

Response: Thanks for your suggestions! In order to present the data of each diagnostic method more clearly, we have added the number of cases and the total number to the data in the Table 1.

Comment 5: Comparison with similar AI-aided methods: The Discussion should be expanded to include a more comprehensive comparison with other AI-aided smear reading systems from recent studies, highlighting the unique advantages and potential limitations of the current system relative to existing technologies.

Response: Thanks for your suggestions! We have expanded the comparison with similar AI-aided methods (TBDx) in Discussion section (Line 248-259) and emphasized both its advantages and limitations of this AI-aided system.

We have highlighted all changes in the revised manuscript. We look forward to your positive response!

Sincerely,

Hairong Huang, MD & PhD

On behalf of the authorship team

Re: Spectrum03970-25R1 (**A Smear Test Aiding with an automated slide reader acquires equivalent sensitivity to MGIT960 culture in Pulmonary Tuberculosis Diagnosis**)

Dear Dr. Hairong Huang:

Your manuscript has been accepted, and I am forwarding it to the ASM production staff for publication. Your paper will first be checked to make sure all elements meet the technical requirements. ASM staff will contact you if anything needs to be revised before copyediting and production can begin. Otherwise, you will be notified when your proofs are ready to be viewed.

Data Availability: ASM policy requires that data be available to the public upon online posting of the article, so please verify all links to sequence records, if present, and make sure that each number retrieves the full record of the data. If a new accession number is not linked or a link is broken, provide production staff with the correct URL for the record. If the accession numbers for new data are not publicly accessible before the expected online posting of the article, publication may be delayed; please contact ASM production staff immediately with the expected release date.

Publication Fees: For information on publication fees and which article types have charges, please visit our website. We have partnered with Copyright Clearance Center (CCC) to collect author charges. If fees apply to your paper, you will receive a message from no-reply@copyright.com with further instructions. For questions related to paying charges through RightsLink, please contact CCC at ASM_Support@copyright.com or toll free at +1-877-622-5543. CCC makes every attempt to respond to all emails within 24 hours.

ASM Membership: Corresponding authors may join or renew ASM membership to obtain discounts on publication fees. Need to upgrade your membership level? Please contact Customer Service at Service@asmusa.org.

Embargo Policy: A press release may be issued as soon as the manuscript is posted on the Spectrum Latest Articles webpage. The corresponding author will receive an email with the subject line "ASM Journals Author Services Notification" when the article is available online.

The ASM Journals program strives for constant improvement in our submission and publication process. Please tell us how we can improve your experience by taking this quick Author Survey.

Thank you for submitting your paper to Spectrum.

Sincerely,
Fei Chen
Editor
Microbiology Spectrum

Reviewer #1 (Comments for the Author):

My comments have been addressed.

Reviewer #2 (Comments for the Author):

The authors have satisfactorily addressed all major concerns raised in the previous round of review.
